# Clinical outcome of revisional gastrectomy postgastric banding failure: A systematic review

**DOI:** 10.1097/MD.0000000000049479

**Published:** 2026-06-26

**Authors:** Mohammad Rashdan, Raha Alzoubi, Alaa Tarazi, Razan Abualrub, Zeina Almanaseer, Hamza Alhashki, Shatha Al Rekabat

**Affiliations:** a Department of General Surgery, Division of Bariatric Surgery, School of Medicine, The University of Jordan, Amman, Jordan; b School of Medicine, The University of Jordan, Amman, Jordan.

**Keywords:** bariatric surgery, clinical outcomes, gastric banding failure, laparoscopic sleeve gastrectomy, revisional gastrectomy, systematic review

## Abstract

**Background:**

Laparoscopic adjustable gastric banding (LAGB) has been widely used for obesity management; however, its long-term failure, due to complications such as band erosion, slippage, and inadequate weight loss, has necessitated revisional bariatric procedures. Revisional laparoscopic sleeve gastrectomy has emerged as a common alternative, yet its safety and efficacy remain debated.

**Methods:**

A systematic review was conducted in accordance with PRISMA guidelines; this review was prospectively registered with Prospective Register of Systematic Reviews (CRD42024568094). A comprehensive search of PubMed, Scopus, Web of Science, and Cochrane databases was performed up to July 19, 2024. Studies published in English that reported postoperative outcomes following revisional laparoscopic sleeve gastrectomy after failed LAGB were included. Data were extracted independently by 2 reviewers, focusing on patient demographics, complication rates (including leaks, bleeding, and infections), and nutritional outcomes. Quality assessment was performed using a standardized tool for observational studies, cohort and cross-sectional studies.

**Results:**

Fifty studies involving a total of 16,192 patients were analyzed. Patients had a mean age between 30 and 50 years and a revision body mass index ranging from 33 to 52 kg/m^2^. Infection was reported in 9 studies, though incidence was low. Bleeding was examined in 30 studies, with mostly isolated cases; thrombosis was reported in only 1 study. Leak rates were reported in 39 studies, ranging from 0% to 8.1%, with stage-specific rates between 2.8% and 5.8%. Four studies noted nutritional deficiencies, including increased ferritin deficiency and some cases contributing to readmission. Overall complication rates were reported in 22 studies, averaging 9.95% and ranging from 0% to 37.5%.

**Conclusion:**

Revisional laparoscopic sleeve gastrectomy following failed LAGB appears to offer a safe and effective alternative, with acceptable complication rates and favorable weight loss outcomes. However, heterogeneity in study designs and patient characteristics underscores the need for standardized reporting and further research to optimize clinical protocols.

## 1. Introduction

Obesity is a growing global health issue, increasingly prevalent due to its association with numerous serious comorbidities. The World Health Organization defines obesity in adults as having a body mass index (BMI) of 30 or higher.^[1]^ Management strategies for obesity typically involve lifestyle modifications, pharmacological treatments, and, in more severe cases, surgical interventions.

Bariatric surgery is commonly indicated for individuals with a BMI ≥ 40, or for those with a BMI ≥ 35 accompanied by obesity-related conditions such as type 2 diabetes, cardiovascular disease, or obstructive sleep apnea.^[2]^ Among the various surgical options, laparoscopic adjustable gastric banding (LAGB) was once considered a safe and effective procedure. It involves placing an adjustable band around the upper portion of the stomach to create a small pouch, thereby restricting food intake and promoting early satiety.

However, over time, LAGB has shown a high rate of failure,^[3]^ with studies reporting suboptimal weight loss outcomes and significant complications such as band slippage and erosion.^[4]^ As a result, the need for revisional bariatric surgeries has increased. A common revisional approach is the conversion to laparoscopic sleeve gastrectomy (RLSG),^[5]^ which involves resecting approximately 80% of the stomach to form a narrow, tube-like structure. This procedure reduces gastric volume, enhances satiety, and supports substantial weight loss.

Despite the rising popularity of RLSG as a revisional option after failed LAGB, concerns remain regarding its safety and efficacy.^[6]^ Conflicting evidence in the literature highlights the need for further evaluation. Therefore, the aim of this systematic review is to assess and synthesize current evidence on the clinical outcomes of RLSG following failed LAGB.

## 2. Methods

### 2.1. Search strategy

A systematic review was conducted in strict adherence to the Preferred Reporting Items for Systematic Reviews and Meta-Analyses (PRISMA) guidelines.^[7]^ The review was prospectively registered with the Prospective Register of Systematic Reviews (PROSPERO) (CRD42024568094). A comprehensive search of the PROSPERO database confirmed the absence of any prior systematic reviews or meta-analyses addressing the same subject matter.

To identify relevant studies, an exhaustive search was performed across 4 databases: PubMed, Scopus, Web of Science, and Cochrane, up to January 22, 2024. The search strategy employed the following terms: (“Sleeve gastrectomy” OR “laparoscopic sleeve gastrectomy” OR “revisional sleeve gastrectomy”) AND (“Gastric banding” OR “Gastric band”) AND (“failure” OR “post” OR “revision” OR “revisional” OR “revisionary”).

Studies included in the review met the following criteria: published in English and focused on a single failed gastric band surgery or a single gastrectomy procedure. Additionally, the studies were required to report postoperative outcomes, with a particular emphasis on complications. We excluded case reports, non-comparative studies, reviews, cadaveric studies, and any articles not published in English.

### 2.2. Screening

The studies were initially screened based on titles and abstracts, followed by a full-text review in accordance with the specified inclusion and exclusion criteria. Two authors independently conducted the search and screening process. Any discrepancies between the reviewers were resolved through discussion with the senior investigator.

### 2.3. Data collection process and data items

By utilizing a standardized Microsoft Office Excel sheet, we were able to extract data from the studies in a consistent manner. Data extraction was conducted by 2 independent reviewers; any disagreements that arose were resolved through discussion and consensus. In addition, information regarding the patients’ demographic characteristics and details of the surgeries were extracted from the included studies such as sample size, age, gender and BMI. Other postoperative outcomes like complication rates, length of hospital stay and weight loss (decrease in BMI, % excess weight loss [EWL] or %excess body mass index loss) were also collected. The quality of the studies was assessed using the Quality Assessment Tool for Observational Cohort and Cross-Sectional Studies to assess aspects such as study population selection, measurement of exposures and outcomes, and statistical analysis. Given that the studies were quite heterogeneous in nature, no meta-analysis was performed, and only the reported data was included and reviewed.

### 2.4. Risk of bias in individual studies

All included studies were observational in nature, and therefore the quality assessment was conducted with the Quality Assessment Tool for Observational Cohort and Cross-Sectional Studies. A number of aspects are covered by this tool; these include study population selection, measurement of exposure and outcome, and statistical analysis. Two reviewers evaluated each study independently on 12 criteria, including sample size justification, exposure assessment, and adjustment for confounding variables. The final quality rating of each study was achieved through a discussion and consensus between the 2 reviewers and a senior author.

## 3. Results

### 3.1. Study selection

**Figure 1.**
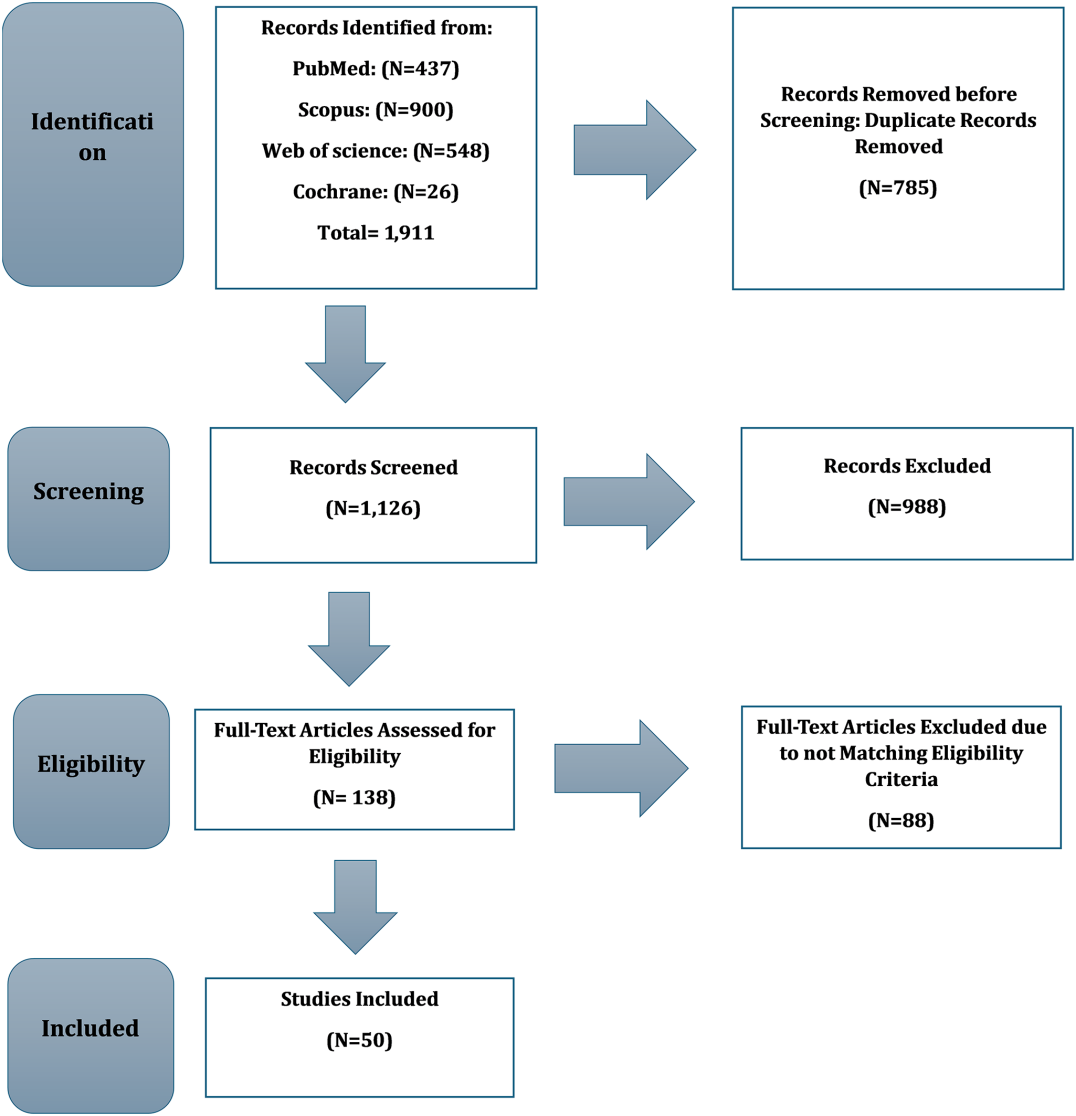
Search strategy flowchart.

Our initial literature search yielded 1911 potentially relevant articles. After removal of duplicate articles, there were 1126 titles and abstracts left for screening. About 988 articles were excluded based on the abstract after an assessment according to our eligibility criteria, leaving 138 for full-text review. Finally, 88 articles were excluded as they did not align review’s main focus leaving 50 articles eligible for inclusion. As shown in Figure 1.

### 3.2. Quality assessment

In evaluating the risk of bias in the included observational studies, the lowest score was 6 out of 12, which was assigned to 2 studies and categorized as poor quality. Conversely, the highest score of 10 out of 12 was achieved by only 2 studies, which were categorized as good quality. The remaining 46 studies scored between 7 and 9 out of 12 and were considered fair quality, as shown in Table 1. The percentage presented in Table 1 represents the proportion of quality criteria fulfilled by each study, with higher percentages indicating better methodological quality and lower risk of bias.

**Table 1:** Quality assessment of the studies.

Studies	Quality score (%)
Antoine et al^[8]^	9/12 = 75%
Moon et al^[9]^	8/12 = 66.7%
Al Sharqawi et al^[10]^	8/12 = 66.7%
Abu-Gazala et al^[11]^	8/12 = 66.7%
Yazbek et al^[12]^	9/12 = 75%
Dapri et al^[13]^	9/12 = 75%
Gagnière et al^[14]^	6/12 = 50%
Bernante et al^[15]^	7/12 = 58.3%
Yeung et al^[16]^	6/12 = 50%
Angrisani et al^[17]^	9/12 = 75%
Creange et al^[18]^	8/12 = 66.7%
Himpens et al^[19]^	8/12 = 66.7%
Demouron et al^[20]^	9/12 = 75%
Rebibo et al^[21]^	9/12 = 75%
Garneau et al^[22]^	8/12 = 66.7%
Thomopoulos et al^[23]^	8/12 = 66.7%
Mendes-Castro et al^[24]^	8/12 = 66.7%
Ngiam et al^[25]^	8/12 = 66.7%
Alqahtani et al^[26]^	7/12 = 58.3%
Carandina et al^[27]^	7/12 = 58.3%
Goitein et al^[28]^	8/12 = 66.7%
Silecchia et al^[29]^	8/12 = 66.7%
Noel et al^[30]^	7/12 = 58.3%
Park & Kim^[31]^	8/12 = 66.7%
Frezza et al^[32]^	7/12 = 58.3%
Kraljević et al^[33]^	8/12 = 66.7%
Angelis et al^[34]^	8/12 = 66.7%
Wickremasinghe et al^[35]^	8/12 = 66.7%
Huang et al^[36]^	10/12 = 83.3%
Xie et al^[37]^	7/12 = 58.3%
Pearlstein et al^[38]^	8/12 = 66.7%
Marin-Perez et al^[39]^	8/12 = 66.7%
Cheema et al^[40]^	7/12 = 58.3%
Barreto et al^[41]^	9/12 = 75%
Patel et al^[42]^	7/12 = 58.3%
Chansaenroj et al^[43]^	8/12 = 66.7%
AlWadaani & Qadeer^[44]^	8/12 = 66.7%
Hany et al^[45]^	9/12 = 75%
Jung Cho & Min Kim^[46]^	8/12 = 66.7%
Gonzalez-Heredia et al^[47]^	9/12 = 75%
Rafols et al^[48]^	7/12 = 58.3%
Santos‐Sousa et al^[49]^	10/12 = 83.3%
Acholonu et al^[50]^	8/12 = 66.7%
Janik et al^[51]^	9/12 = 75%
Spaniolas et al^[52]^	7/12 = 58.3%
Foletto et al^[53]^	7/12 = 58.3%
Dowgiałło‐Gornowicz et al^[54]^	8/12 = 66.7%
Khan et al^[55]^	7/12 = 58.3%
Khoursheed^[56]^	7/12 = 58.3%
Falk et al^[57]^	8/12 = 66.7%

### 3.3. Patient and studies characteristics

A total of 16,192 patients were included in this study. The majority of the studies showed a female predominance over males. The mean age of participants ranged from 30 to 50 years. Most of the included studies had a follow-up period of at least 6 months. The mean BMI at the time of revision ranged from 33 to 52 kg/m^2^. Additional study characteristics are presented in Table 2.

**Table 2:** Studies characteristics.

Study	Number of participants	Age (mean)	Gender	Follow-up	BMI at revision (mean)	BMI before band (mean)	Time to revision	Long-term complications	Other complications
Antoine et al^[8]^	23	44.1 (22–71)	Males only (23)	2 yr	42.2	45.4	89.8 mo	-GERD (2.7%)-incisional hernia repair (1%).	hematoma and stenosis
Moon et al^[9]^	13	41.0	M:F 1:12	24 mo	39.0	41.0	39.5 mo	N/A	N/A
Sharqawi et al^[10]^	40	36	M:F 6:34	6 mo to 3 yr (median, 1 yr)	42	44	4 yr	N/A	Insufficient weight loss: 4 Persistent vomiting: 2 Biliary colic: 2 Staple line leak: 0 Stricture: 0 Reflux: 2
Abu-Gazala, et al^[11]^	18	38.6	M:F 4:14	14 +/-11.6	40	N/A	N/A	N/A	Intra-abdominal abscess
Yazbek et al^[12]^	90	41	M:F 13:77	2 yr (6 mo –4 yr)	42	N/A	40 mo	N/A	abdominal adhesions:2 colonic perforation: 1 splenic laceration: 1 sleeve strictures: 2
Dapri, et al^[13]^	27	43.6	M:F 10:17	18.6 mo (range 1–59)	39	45	51.2 mo (range 22–132)	N/A	N/A
Gagnière et al^[14]^	31	43.1	N/A	N/A	40.6	N/A	6 mo	N/A	- Pleuropneumonia 1 -Superficial surgical site abscess 1 -Dysphagia 2 -Intractable abdominal pain 2 -Pyrosis 3
Bernante, et al^[15]^	8	46.6	M:F 2:6	12 mo	50.5	N/A	2 to 13 yr	N/A	N/A
Yeung et al^[16]^	72	44.9	M:F 11:61	12 mo	39.63	N/A	6 yr	1 incarcerated port-site hernia	Aspiration pneumonia, Acute angle glaucoma, Stricture
Angrisani et al^[17]^	27	37.5	N/A	5 yr	39.7	N/A	75.7 mo	N/A	N/A
Creange et al^[18]^	283	43.2	M:F 79:204	5 yr	43.0	46.5	N/A	N/A	N/A
Himpens et al^[19]^	40	47.2	M:F 17:23	32.6 mo	43.5	46	60.2 mo	Incisional hernia: 1	N/A
Demouron et al^[20]^	358	1-step: 44.0 2-step: 44.3	M:F 40:318	24 mo	1-step: 40.5 2-step: 43.5 (31.5–61.7)	1-step: 44.2 2-step:46.7	10 yr	N/A	Stenosis 1-step: 2 Others 2-step: 1
Rebibo et al^[21]^	46	42	M:F 3:43	24 mo	44	N/A	48 mo	N/A	gastric fistula:2 gastric stenosis: 1 gastric stenosis + collection in pouch of Douglas: 3
Garneau et al^[22]^	75	46	M:F 14:61	1–3 yr	45	N/A	40 mo	N/A	Stenosis:1
Thomopoulos et al^[23]^	76	49	M:F 62:14	4.3 yr	46.7	N/A	42 mo	Ten patients (10%) had long-term complications (8 severe reflux and 2 stenosis)	N/A
Mendes-Castro et al^[24]^	17	47.1	M:F 5:12	4.3 mo	42.2	47	7.6 yr	N/A	N/A
Ngiam et al^[25]^	6	45.7	M:F 4:2	54 mo	N/A	35.2	N/A	N/A	N/A
Alqahtani et al^[26]^	56	33.5±	M:F 17:39	2 yr	44.4	47.9	N/A	N/A	o
Carandina et al^[27]^	100	41.1	M:F 15:85	7 yr	44.4	47.9	N/A	N/A	Stenosis 3%
Goitein et al^[28]^	46	40	M:F 12:34	17 mo	43.1	N/A	2 yr	N/A	N/A
Silecchia et al^[29]^	76	45.5	M:F 16:60	24 mo	43.9	N/A	51 mo		Anemia and transient food intolerance = 9 Transient dysphagia, leukocytosis, fever, and anemia = 4
Noel et al^[30]^	300	43	M:F 76:224	60 mo	43	N/A	3 mo at least	N/A	Stenosis: 2
Park & Kim^[31]^	9	34.7	All females	19.1 mo	34.0	40.4	minumim 3 mo	N/A	Stenosis: 1
Frezza, et al^[32]^	10	N/A	M:F 3:7	12–32 mo	39–56	N/A	N/A	N/A	Reflux: 1
Kraljević et al^[33]^	45	41.6	M:F 14:36	7.5 yr	46.3	N/A	N/A	reflux (32.4%, 1.5% combined with hiatal hernia) incisional hernia (4.9%), stenosis (1.3%), and late leak at the staple line (0.3%). Mortality related to LSG was zero	N/A
Angelis et al^[34]^	44	46	M:F 10:34	9.3 yr	42.51	N/A	N/A	type I hiatal hernia: 5	At the end of the follow-up, hypertension was present in 18.1% of the revisional and type II diabetes in 2.3% GERD
Wickremasinghe et al^[35]^	600	M:F 522:78	45	12 mo	43.8	N/A	3 mo	Stenosis: 0 Fistula: 2	Wound problems 4 Bands strictures: 1 Dysphagia:4 Nausea: 2 vomiting:2 pan esophageal dilatations stricture:2 tachycardia:1 fever: 1 wound dehiscence: 1 (leading to a significantly longer length of hospital stay (3.9 ± 2.4 vs 2.9 ± 2.1 days, *P*-value 0.025))
Huang et al^[36]^	72	45.3	M:F 10:62	18 mo	42.7	N/A	3.8 (2.1–57.4) mo	Hiatal hernia repair was performed in 37 (51.4%) RLSG cases	One RLSG patient developed severe nausea and vomiting, One RLSG patient had a stricture at the previous band tunnel One other RLSG patient also underwent revision gastric bypass after 1.7 and due to intractable reflux.
Xie et al^[37]^	131	44.3	M:F 8: 123	2 weeks, 1 mo, every 3 mo for the first 2 yr, and then annually.	45.6	N/A	88.3 mo	Ventral hernia 7 (3.2%) Petersen hernia 2 (0.9%) Small bowel obstruction 2 (0.9%)i	1 Pulmonary embolism/ 1 intraoperative complication (splenic vessel injury) 3 splenic vessel injury and adhesions
Pearlstein et al^[38]^	40	44.9	M:F 9:31	12 mo	47.7	N/A	N/A	Incarcerated hernia and bowel perforation	N/A
Marin-Perez et al^[39]^	59	44	M:F 11:48	33 mo	39	45	31 mo	Large paraoesophageal hernia: 1 small bowel resection: 2 marginal ulcer with bleeding: 1	dilatation of the sleeve proximal to distal compression, which resolved with operative adhesiolysis: 1 acute complete obstruction of the proximal gastric sleeve that required conversion to LRYGB after 2 days: 1
Cheema et al^[40]^	37	44.2	M:F 5:32	2 yr	45.1	N/A	9.1 mo	4 grade IIIb complications, requiring an intervention under general anesthesia, drainage of intra-abdominal abscess (AGB to SG)	N/A
Barreto et al^[41]^	76	50	M:F 11:65	2 yr	N/A	N/A	N/A	N/A	Aspiration pneumonia—1 patient Superior mesenteric vein thrombus-1 patient
Patel et al^[42]^	12	N/A	N/A	2 yr	35.8	N/A	26.8 mo	Reflux/ulcer disease: 3	Two patients experienced perioperative complications. 1 patient had an intraoperative perforation of the colon with an Optiview trocar
Chansaenroj et al^[43]^	17	42.8	M:F 9:8	2 yr	33.8	34.6	2 yr	N/A	0
AlWadaani & Qadeer^[44]^	36	30.67	M:F 6:30	24 mo	43.50	N/A	36.83 mo	N/A	Seven patients though initially reduced the weight after RLSG, but afterwards they either failed to reduce further or started gaining weight around the period of 2 yr. This was observed more in those patients who were frequent sweet eaters and/or not doing/stopped regular exercise
Hany et al^[45]^	81	43.6	M:F 20:61	2 yr	44.1	50.5	3.6 yr	Port-site hernia:1	GERD Grade A:1 Hiatal hernia:4 Helicobacter Pylori: 7 Dilated esophagus: 12
Jung Cho & Min Kim^[46]^	6	35	N/A	33.8 mo	32.7	33.6	15.7 mo	N/A	N/A
Gonzalez-Heredia et al^[47]^	26	38.6	N/A	12 mo	48.6	N/A	59.7 mo	N/A	0
Rafols et al^[48]^	123	38.2	M:F 29:94	33 mo	41.4	45.6	N/A	N/A	N/A
Santos‐Sousa et al^[49]^	20	51.0	M:F 3:17	12 mo	44.63	N/A	N/A	N/A	N/A
Acholonu et^[50]^	15	46.6	M:f 12:3	24 mo	38.66	N/A	34.7 mo	N/A	-Band slippage and GERD: 4 -Slippage and duodenal fistula: 1
Janik et al^[51]^	9192	48.27	M:F 1566: 7625	30 days	42.03	N/A	N/A	Strictures/stomal obstruction: 7 Intestinal obstruction: 2 Abdominal pain: 1 GI perforation: 1 Anastomotic/Staple line leak: 17 Bleeding: 13 Other abdominal sepsis: 5 Wound infection/evisceration: 4 Nausea and vomiting, fluid, electrolyte, or nutritional depletion: 2	Other: 25 Abdominal Pain, Not Otherwise Specified: 21 Pulmonary Embolism: 5
Spaniolas et al^[52]^	3364	47.2	M:F 525: 2839	30 days	42.2	N/A	N/A	N/A	N/A
Foletto et al^[53]^	57	49.9	M:F 20:37	20 mo	45.7	51.2	7.54 yr	Large incisional hernia: 3 complicated paraesophageal hernia: 1	Dysphagia: 4 mortality: 3 (1: diffuse peritonitis, 2; COPD exacerbation, 3: pulmonary embolism)
Dowgiałło‐Gornowicz et al^[54]^	116	47.7	M;F 44:72	23 mo	40.6	43.9	5.7 yr	N/A	Ileus: 6.0%, GERD: 14.7%
Khan et al^[55]^	23	38.0	N/A	2.2 yr	40.3	50.5	75 mo	N/A	0
Khoursheed et al^[56]^	42	35.6	M:F 6:36	9.8 mo	38.5	N/A	N/A	0	0 Vanessa Falk
Falk et al^[57]^	30	46.6	M:F 5:25	1 yr	44.3	45.6	4.5 yr	0	1 Laparoscopy for drain placement and jejunal feeding tube 1 endoluminal stent Laparoscopy for abnormal Gastrografin swallow study (POD 1)

EWL = excess weight loss, F = female, GERD = gastroesophageal reflux disease, M = male, N/A = not applicable.

### 3.4. Weight loss

Across 38 studies, RLSG after failed gastric banding was associated with favorable weight loss outcomes. Most reports highlighted significant reductions in BMI, %EWL, and percentage of total weight loss (%TWL), though these outcomes varied by surgical plan and the duration of follow-up. EWL of 50% average or greater, which is commonly used as a clinical benchmark for successful weight loss, was achieved in 11 reports.^[8–10,15,21,24,27,28,30,32,38]^ Huang et al reported that Primary LSG (PLSG) showed higher %EWL than RLSG at 3 to 36 months, but differences became insignificant by 48 to 60 months.^[36]^ Another study by Cheema et al reported significantly lower %EWL in patients who underwent RSG than in patients who had undergone PSG.^[40]^ However, Ngiam et al reported that there was no difference in the weight reduction trajectories between primary LAGB and revisional surgeries at 5 years.^[25]^

Notably, 3 studies used the percentage of total body weight loss to represent weight loss outcomes instead of %EWL.^[35,37,41]^ Barreto et al showed that weight loss outcomes following conversion of LAGB to LSG or RYGB were significantly different, as patients who underwent revision to LSG had significantly lower %TWL at 1, 3, and 5 years compared with those revised to RYGB.^[41]^ Similarly, Wickremasinghe et al reported a mean total body weight loss of 22.9% for those who underwent RLSG, compared to 29.6% in the primary sleeve group.

Demouron et al reported no significant difference in %EWL between 1-step (40.5–33.8 kg/m^2^) and 2-step (43.5–34.6 kg/m^2^) approaches at 24 months.^[20]^ However, weight loss tended to plateau between 12 and 18 months,^[33,34]^ as Kraljević et al and de Angelis et al observed in their studies a sustained BMI reduction beyond 5 years, with an average EWL of 53 ± 26% follow-up at 8 years.^[34]^

### 3.5. Infection

Eight studies reported infection as a complication following RLSG,^[25,26,35–37,44,45,47]^ although the overall incidence of infection was low. Two studies found no infections among their participants post-surgery.^[25,44]^ Four studies reported a single infection in 1 patient each after revisional LSG.^[26,35–37]^ The highest incidence involved 4 reported cases of infection across 2 studies.^[44,47]^

### 3.6. Bleeding and thrombosis

Regarding bleeding complications, 30 of the included studies addressed this issue, with a relatively consistent incidence across them. Seven studies reported no hemorrhage after RLSG.^[10,15,26,29,43,45,47]^ Additionally, the study by Hani et al identified 2 cases of bleeding without the need for blood transfusion.^[26]^ Six studies reported 1 case of hemorrhage each.^[19,21,27,28,30,37]^

Several studies mentioned the occurrence of gastric and parietal hematomas. Yazbek et al reported 4 cases of gastric hematoma and 2 cases of parietal hematoma.^[12]^ Gagniere et al documented 1 case of intra-abdominal hematoma and 1 case of hemoperitoneum.^[14]^ Two studies reported patients with only gastric hematomas (2 and 1 hematoma, respectively),^[22,56]^ while Khoursheed et al reported 2 cases of wound hematomas.^[56]^ Additionally, Dapri et al described 1 case of subphrenic hematoma that required laparoscopic drainage on the 2nd postoperative day.^[13]^ Foletto et al reported 3 cases of perigastric hematomas.^[53]^

Three studies reported 2 cases of bleeding each.^[35,44,48]^ The study by Antoine et al noted 4 cases of bleeding and 1 case of hematoma.^[9]^ Furthermore, bleeding occurred in 0.9% of patients in 1 study^[54]^ and 1% in another.^[51]^ Marin-Perez et al reported an average blood loss of 74 mL among RLSG patients.^[39]^ Two studies compared hemorrhage rates between 1-stage and 2-stage RLSG procedures: at stage 1, bleeding occurred in 1.5% and 2.8% of patients, while at stage 2, the incidence was 2.2% and 4.3%, respectively.^[20,36]^

In contrast, only 1 study reported thrombosis as a complication. Kraljevic et al documented 1 case of portal vein thrombosis and 1 case of splenic infarction, both of which were treated conservatively.^[33]^

### 3.7. Leak

Regarding leaks as a complication following RLSG, 40 studies reported on this outcome. Among them, 12 studies reported no leaks among their participants.^[10,13,15,25,26,29,38,43–46,56]^ Seven studies each reported a single case of leak,^[11,16,22,39,42,46,50]^ with one of these studies noting that the patient required reoperation.^[42]^ Another study by Xie et al reported a case of staple line leak requiring percutaneous drainage, along with additional patients who needed reoperation.^[37]^

Two studies examined leak outcomes at 2 stages: Demouron et al reported a leak rate of 3.7% at step 1 and 3.4% at step 2,^[20]^ while Huang et al reported leak rates of 5.8% at stage 1 and 2.8% at stage 2.^[36]^

The remaining studies that reported leaks^[8,11,16,18,27,28,30,31,33,35,48,51,52,54,55,57]^ showed a range of leak incidences from 0.3%^[12,22]^ to 8.1%.^[48]^

### 3.8. Nutrition

For the nutritional status of patients after RLSG, 4 studies reported on this outcome. The study by Noel et al noted nutritional deficiencies in 3 patients who underwent RLSG.^[35]^ Another study reported the mean hemoglobin levels at 1 and 2 years of follow-up, which were 15.9 and 15.1 g/dL, respectively.^[43]^ Hany et al observed hemoglobin deficiency in 19 RLSG patients. The mean food tolerance after 1 and 2 years of follow-up was 21.5 and 22.2, respectively. They also reported no significant changes in nutritional deficiencies after 2 years, except for a higher rate of ferritin deficiency post-surgery (10% compared to 0% pre-surgery).^[45]^ Additionally, 1 study highlighted that nausea, vomiting, and fluid, electrolyte, or nutritional depletion contributed to readmission in 45 patients and reoperation in 2 RLSG patients.^[51]^

### 3.9. Complication rate

In regard of the overall complication rate, 22 studies reported this outcome. The mean complication rate across these studies was approximately 9.95%. Two studies reported a 0% complication rate following RLSG.^[43,47]^ Demouron et al reported the overall complication rates for patients who underwent the procedure at step 1 and step 2, with rates of 11.1% and 10.2%, respectively.^[20]^ The lowest complication rate, aside from the 0 complication studies, was 2%, reported by Noel et al.^[30]^ The remaining studies had complication rates falling within this range, from the lowest to the highest.^[11,16,17,21,23,27–29,36–38,45,48,49,51,54,56,57]^

## 4. Discussion

Worldwide obesity rates have risen sharply over the past few decades, leading to negative health consequences such as diabetes mellitus, obstructive sleep apnea, stroke, coronary heart disease, hypertension, and gastroesophageal reflux disease, highlighting obesity as a major public health concern.^[58]^ Bariatric surgery is the most effective long-term treatment for severe obesity, not only resulting in significant and sustained weight loss but also improving many obesity-related comorbidities.^[58,59]^ Since its emergence in the mid-90s, LAGB has become increasingly favored by obese patients due to its advantages in simplicity, rapid recovery, and reversibility. However, it’s no longer commonly performed due to the recurrent occurrence of complications, such as pouch dilation, stoma obstruction, and band erosion, which may lead to revisional surgeries.^[59,60]^ Although it is controversial whether another restrictive surgery should be done after gastric banding failure due to band erosion or any other complication, many patients seek revisional LSG.^[60]^ In this article, we discuss the clinical outcomes of LSG post-failed LAGB.

Substantial clinical and methodological heterogeneity precluded meta-analysis. All included studies were observational and demonstrated marked variability in patient characteristics, revision BMI, surgical approach (e.g., 1-stage vs 2-stage conversion), follow-up duration, and outcome reporting. Complication definitions and weight loss metrics were inconsistently reported across studies. Reported overall complication rates ranged from 0% to 37.5%, and leak rates from 0% to 8.1%, reflecting differences in case selection and operative techniques. Pooling such heterogeneous data could produce a statistically precise yet clinically misleading estimate; therefore, a narrative synthesis was considered more appropriate. Clinically, these findings should be interpreted as a range of reported outcomes rather than a single expected complication rate. Standardized reporting in future studies is necessary to enable meaningful quantitative synthesis and improve generalizability.

### 4.1. Weight loss

Although RLSG after failed LAGB was associated with significant reductions in BMI, %EWL, and %TWL across the included studies, our findings do not demonstrate the superiority of RLSG over PLSG. Importantly, several comparative studies suggested that weight loss outcomes following revisional sleeve gastrectomy may be inferior to those achieved with primary procedures, particularly in the short- and mid-term follow-up periods. The variability in outcomes between the studies may be due to multiple factors such as variations in surgical technique (e.g., single-stage vs 2-stage revision), the time of each operation, patient past medical history (the more chronic diseases a patient has, the less weight loss), preoperative BMI, and differences in follow-up durations. Short-term follow-up data reveal more successful weight loss results; on the other hand, longer-term data are more likely to show difficulty in weight maintenance and complications. In addition to that, conversion of LAGB to LSG or RYGB was significantly different, as patients who underwent revision to LSG had significantly lower percent TWL at 1, 3, and 5 years compared with those revised to RYGB. This can be due to mechanisms of action associated with each procedure.^[61]^ SG involves removing a large portion of the stomach at the gastric fundus and greater curvature of the stomach. This limits the size of the stomach, producing a tubular conduit. With RYGB, however, a small pouch is created from the proximal stomach; this pouch is connected to a loop of jejunum, creating a gastrojejunostomy. The remainder of the stomach and proximal small bowel is left intact and re-anastomosed distal to the gastrojejunostomy, thus remaining isolated from digestive flow. So, SG is restrictive, while RYGB combines both restrictive and malabsorptive components, potentially leading to greater weight loss.^[62]^

Taken together, these findings indicate that while RLSG is a feasible and generally safe revisional strategy, it should not be considered superior to primary sleeve gastrectomy in terms of weight loss. Preoperative counseling should therefore include realistic expectations regarding achievable weight reduction after revisional surgery.

The wide variability in postoperative weight loss following RLSG has important clinical implications. These findings suggest that outcomes are highly patient-dependent and influenced by surgical technique, baseline metabolic status, and follow-up duration. Therefore, RLSG should not be viewed as a uniform solution, and careful patient selection and preoperative counseling are essential.

In addition, the rapid evolution of pharmacologic obesity therapy warrants consideration. Highly effective agents such as semaglutide and liraglutide, as well as the dual GLP-1/GIP agonist tirzepatide, have demonstrated substantial weight loss, in some cases approaching surgical outcomes.^[63]^ This development raises important questions regarding the necessity of revisional surgical procedures in selected patients.

While surgery may still provide durable weight loss and comorbidity improvement in appropriately selected individuals, the choice between revisional surgery and advanced pharmacotherapy should be individualized within a multidisciplinary framework.

### 4.2. Infection

The incidence of infection following RLSG appears to be relatively low based on the reviewed studies. Eight studies reported infections as a postoperative complication. Among these, 2 studies noted no infections among their participants, while 4 studies reported a single infection case each. The highest infection rates were found in 2 studies, each reporting 4 cases.

Variations in infection rates can be attributed to both patient-related (intrinsic) and procedural (extrinsic) factors. Intrinsic factors include comorbidities, age, gender, history of previous surgeries, and BMI. For example, patients with a normal BMI (18.5–24.9 kg/m^2^) tend to have lower rates of surgical site infections (SSIs), while a BMI over 30 is associated with a higher risk, particularly in patients undergoing revisional surgery, who typically present with elevated BMI.

Age may also play a role, though findings are inconsistent. Some studies report higher infection rates in older patients, while others indicate lower rates in patients aged 55 and older compared to younger individuals.^[64]^ Smoking is another known risk factor, as it negatively affects capillary oxygenation and tissue perfusion, impairing wound healing.^[65]^

Gender differences may also influence infection risk. In men, androgens can exert a pro-inflammatory effect that delays wound healing, whereas in women, estrogens appear to have anti-inflammatory and healing-promoting effects.^[66–68]^

The use of postoperative drains has been identified as a significant risk factor for infection, likely due to the potential for bacterial colonization.^[69]^ The primary sources of SSIs are often endogenous, stemming from bacterial flora on the skin or in the alimentary and genital tracts.^[70]^ *Staphylococcus aureus* is the most commonly isolated organism. Though less common, exogenous sources, such as lapses in sterile technique or contaminated equipment, can also contribute.^[71]^

Bacterial presence within tissue or organ spaces can hinder healing, potentially leading to complications such as anastomotic leaks, wound dehiscence, and superficial incisional infections.^[72]^ Although infections are relatively uncommon, they remain a serious concern due to their impact on recovery and patient outcomes.

To mitigate infection risks, healthcare institutions and surgical teams must adopt stringent infection control protocols, follow evidence-based guidelines, and ensure comprehensive preoperative evaluation, optimization of patient health, and attentive postoperative care. These measures are essential for reducing the incidence of SSIs and enhancing overall surgical outcomes.^[73]^

### 4.3. Bleeding and thrombosis

According to the results of the included studies, bleeding complications following RLSG are relatively rare. However, when they do occur, they can lead to significant clinical deterioration and must be taken seriously.^[74]^ Prompt diagnosis and management are crucial, requiring a high index of suspicion. A multidisciplinary team approach, encompassing surgeons, endoscopists, and radiologists, is strongly recommended to ensure effective management of complex cases.^[75]^

While 8 studies reported no hemorrhagic events, a small but consistent number of studies documented variable incidences of bleeding, hematomas, and, in rare cases, thrombosis. Most bleeding events reported were minor, self-limiting, and did not necessitate blood transfusions, indicating a generally low overall incidence of clinically significant hemorrhage.

Understanding the potential causes of postoperative bleeding is essential. Surgical technique errors, particularly in revisional procedures, pose a heightened risk due to altered anatomy, scarring, and adhesions from previous surgeries.^[76]^ Additionally, undiagnosed preexisting conditions, such as coagulation disorders or gastric ulcers, may increase the likelihood of postoperative bleeding.^[77]^

In the study by Hani et al, 2 cases of bleeding were identified, neither requiring transfusion. Similarly, 6 other studies reported only a single hemorrhagic event each. These findings suggest that most bleeding complications are manageable without invasive interventions. Nevertheless, 1 study documented a bleeding incidence of up to 1%, indicating some variability. This variation may be due to differences in surgical technique, patient populations, and inconsistent reporting methodologies across studies.

Hematomas following RLSG were also infrequent. Yazbek et al reported the highest number, documenting 4 gastric and 2 parietal hematomas. Other studies described isolated cases of intra-abdominal, subphrenic, and wound hematomas. Most were self-limiting and did not require surgical intervention; only 1 case necessitated laparoscopic drainage. While generally benign, hematomas may occasionally demand medical attention, especially if they increase in size or present signs of infection such as erythema, warmth, or fever.^[78]^

It is also noteworthy to compare hemorrhage rates between 1-stage and 2-stage RLSG procedures. Slightly higher bleeding rates were reported in the second stage of 2-stage procedures, ranging from 2.2% to 4.3%, compared to 1.5% to 2.8% in the first stage. These differences may reflect increased procedural complexity and altered tissue characteristics in staged surgeries. While the 2-stage approach may offer benefits such as reduced risk in high-risk patients or a more controlled operative environment, the second stage requires careful surgical technique to minimize bleeding risk. Ultimately, the choice between 1-stage and 2-stage RLSG depends on individual patient factors, surgical expertise, and clinical judgment.^[79]^

Thrombotic complications appear to be exceedingly rare. Only 1 study reported such an event, detailing a case of portal vein thrombosis and splenic infarction, both of which were managed conservatively. Despite their rarity, thrombotic events warrant attention due to their potentially serious consequences.

In summary, bleeding and thrombotic complications following RLSG are uncommon and usually manageable. However, the observed variability in incidence underscores the need for standardized reporting criteria and further research to better understand the contributing factors. Clear perioperative protocols and vigilant postoperative monitoring remain essential to optimize patient safety and outcomes.

### 4.4. Leak

Leakage following RLSG is a significant and well-documented postoperative complication. The findings of this review are consistent with existing literature, which indicates that leak rates vary considerably among patients and surgical settings. Of the 51 studies included in the analysis, several reported no instances of leaks, suggesting that RLSG can often be performed safely and without this complication in many cases.^[74]^ However, other studies documented single leak events, some of which necessitated reoperation.^[75]^ Staple line leaks have previously been managed through interventions such as percutaneous drainage or surgical reintervention, depending on the severity and clinical presentation.^[76]^

The occurrence of leaks is influenced by multiple factors, including patient-specific characteristics (e.g., high BMI, history of previous bariatric surgery), the technical proficiency of the surgeon, and measures taken during preoperative and intraoperative periods.^[74]^ For example, Demouron et al found that leak rates were slightly lower in the second stage of a staged RLSG procedure (3.4%) compared to the first stage (3.7%). Similarly, Huang et al observed a notable reduction in leak rate from 5.8% in stage 1 to 2.8% in stage 2, suggesting that improved tissue adaptation and reduced tension on the staple line in staged approaches may contribute to lower leak incidence.^[75]^

Nonetheless, leak rates continue to vary across studies. This variability is likely due to differences in patient demographics, surgical techniques, perioperative care protocols, and institutional practices.^[77]^ These discrepancies highlight the need for standardized prevention strategies to minimize leak risk. Recommended measures include staple line reinforcement, careful patient optimization before surgery, and the use of routine postoperative imaging, such as contrast swallow studies or early CT scans, for high-risk patients.^[78]^

Notably, staged 2-stage RLSG procedures demonstrated a safety advantage in several studies, with lower leak rates observed during the second stage. This reduction may be due to improved tissue quality, resolution of inflammation, and decreased tension on the staple line, supporting the potential benefit of a staged approach in selected high-risk patients. However, this advantage must be weighed against a slightly increased risk of bleeding in the second stage, likely resulting from adhesions, altered vascular anatomy, and technical complexity. Strategies to mitigate bleeding include meticulous adhesiolysis, careful hemostasis, staple line reinforcement, and thorough preoperative assessment of coagulation status.^[80]^ These findings highlight the need for individualized operative planning and careful patient selection when considering staged RLSG.

### 4.5. Nutrition

Long-term success after weight reduction surgeries like RLSG depends heavily on maintaining a healthy diet.^[81]^ In our review, 4 studies addressed the nutritional status of patients post-RLSG, highlighting various deficiencies and related complications.

Noel et al reported nutritional deficiencies in 3 patients, suggesting that while RLSG is effective for weight loss, it may compromise nutrient intake, particularly in patients undergoing RLSG after failed LAGB, who demonstrated poorer eating habits compared to those following PLSG.^[81,82]^ Also, the study by Chansaenroj P et al monitored hemoglobin levels, noting a decline from 15.9 g/dL at 1 year to 15.1 g/dL at 2 years, indicating a potential risk of anemia.^[83]^

Hany et al found hemoglobin deficiency in 19 patients and evaluated food tolerance post-surgery. The average food tolerance score was 21.5 after 1 year, improving slightly to 22.2 by year 2, suggesting gradual dietary adaptation. However, a notable increase in ferritin deficiency was observed, rising from 0% preoperatively to 10% postoperatively, highlighting a growing risk of iron deficiency, which can lead to fatigue, weakness, and other health issues.^[84]^

Additionally, 1 study reported that nausea, vomiting, and fluid or electrolyte imbalances were significant postoperative complications, resulting in 45 hospital readmissions and 2 reoperations. These findings emphasize the importance of ongoing nutritional monitoring and management to support recovery and prevent long-term health complications.^[85]^

RLSG can significantly alter gastrointestinal morphology, which may predispose patients to malabsorption of macronutrients and micronutrients, including proteins, carbohydrates, fats, vitamins, and minerals. Although only a few studies in this review systematically reported nutritional outcomes, deficiencies such as iron, ferritin, and hemoglobin were observed, along with symptoms like nausea, vomiting, and poor food tolerance. These findings underscore the potential for moderate-to-severe nutritional compromise in the postoperative period. Therefore, intensive, long-term monitoring of nutritional status is essential, including regular laboratory assessment, dietary counseling, and supplementation as indicated. Early identification and correction of deficiencies are crucial to prevent complications such as anemia, fatigue, and other metabolic disturbances and to ensure the sustained success of revisional bariatric procedures.

### 4.6. Complication rate

A total of 22 studies assessed the overall complication rate following RLSG, offering valuable insights into the risks associated with the procedure. The average complication rate across these studies was approximately 9.95%, indicating that while complications do occur, they are relatively infrequent for most patients.^[86]^

Two of the included studies reported a 0% complication rate, meaning no postoperative complications were observed in those cohorts. However, complication rates varied across the remaining studies. For example, Demouron et al compared 1-stage and 2-stage procedures, reporting complication rates of 11.1% and 10.2%, respectively, suggesting slightly better outcomes in staged surgeries.

Noel et al reported one of the lowest non-zero complication rates at 2%, while other studies reported a higher different incidence of this outcome, underscoring the variability in outcomes that may be influenced by patient profiles, surgical expertise, and institutional practices.^[86]^

The remaining studies reported complication rates ranging between 2% and 37.5%, reflecting the broad spectrum of clinical scenarios. This variation highlights the need for consistent monitoring, standardization of surgical techniques, and individualized risk assessment to improve postoperative outcomes and enhance patient safety.^[87,88]^

## 5. Conclusion

This systematic review demonstrates that sleeve gastrectomy following failed gastric banding is generally safe, with a low incidence of major complications. Infection rates were minimal, and bleeding events were uncommon, typically requiring little to no intervention, with only a few reported cases of hematomas or thrombosis. Leak rates were also low, ranging from 0.3% to 8.1%, though variability across studies suggests an influence of patient factors and surgical technique. Nutritional complications, particularly iron and ferritin deficiencies, can be clinically significant and may impact patient well-being and performance, underscoring the importance of intensive, long-term postoperative monitoring and supplementation when indicated. The overall complication rate averaged 9.95%, with reported rates ranging from 0% to 37.5%, highlighting the need for standardized surgical protocols and patient optimization to minimize risks.

## 6. Limitation

This review has several limitations. First, the included studies exhibited substantial heterogeneity, which prevented the performance of a meta-analysis. Second, the review was limited to English-language publications and included only cohort studies, making the findings dependent on the accuracy and completeness of follow-up data. Third, many studies lacked sufficient detail to fully assess methodological quality, potentially affecting the overall reliability of the review. Future research should aim to include more homogeneous and higher-quality studies to strengthen the evidence base.

## Author contributions

**Conceptualization:** Mohammad Rashdan, Raha Alzoubi, Alaa Tarazi.

**Data curation:** Raha Alzoubi, Alaa Tarazi, Razan Abualrub, Zeina Almanaseer, Hamza Alhashki, Shatha Al Rekabat.

**Formal analysis:** Alaa Tarazi.

**Investigation:** Raha Alzoubi, Alaa Tarazi, Razan Abualrub, Zeina Almanaseer, Hamza Alhashki, Shatha Al Rekabat.

**Methodology:** Raha Alzoubi, Alaa Tarazi, Razan Abualrub, Zeina Almanaseer.

**Project administration:** Alaa Tarazi.

**Resources:** Alaa Tarazi, Razan Abualrub.

**Supervision:** Mohammad Rashdan.

**Validation:** Mohammad Rashdan.

**Writing – original draft:** Raha Alzoubi, Alaa Tarazi, Razan Abualrub, Zeina Almanaseer, Hamza Alhashki, Shatha Al Rekabat.

**Writing – review & editing:** Mohammad Rashdan, Raha Alzoubi, Alaa Tarazi, Razan Abualrub, Zeina Almanaseer, Hamza Alhashki, Shatha Al Rekabat.
